# Supplementary material for: Enhancing geometric representations for molecules with equivariant vector-scalar interactive message passing
Source: Nat Commun. 2024 Jan 5;15:313. doi: 10.1038/s41467-023-43720-2 (PMC10770089; doi:10.1038/s41467-023-43720-2)
Supplement: Supplementary file 1 — Supplementary material [file 41467_2023_43720_MOESM1_ESM.pdf]

Supplementary Information for
Enhancing geometric representations for molecules with equivariant vector-scalar interactive message passing

Supplementary Methods

1.1 Embedding Block

The edge distance is filtered through linear combination of radial basis function (RBF):

$$\mathrm{g}_k(\vec{r}_{ij}) = \phi(\|\vec{r}_{ij}\|) \cdot \exp\left(-\beta_k \left(\exp\left(-\|\vec{r}_{ij}\|\right) - \mu_k\right)^2\right) \tag{1}$$

where β_k, μ_k are optional learnable parameters that specify center and width of $\mathrm{g}_k(\vec{r}_{ij})$, and $\phi(\cdot)$ is a smooth cosine cutoff function.

Then, the extended edge embedding is given by:

$$e_{ij}^0 = W_e\left(\mathrm{g}(\vec{r}_{ij})\right) \tag{2}$$

The neighborhood embedding is defined as:

$$n_i = \sum_{j \in \mathcal{N}(i)} \mathrm{embed}^{\mathrm{nbh}}(z_j) \odot W_f(\mathrm{g}(\vec{r}_{ij})) \tag{3}$$

with $\mathrm{embed}^{\mathrm{nbh}}(\cdot)$ denoting the neighbor embedding function. If W_e and W_f shares the same weight, it represents we directly combine the edge embedding in generating the node embedding.

The initial node embedding is given by:

$$h_i^0 = W_h[\mathrm{embed}^{\mathrm{int}}(z_i), n_i] \tag{4}$$

with $\mathrm{embed}^{\mathrm{int}}(\cdot)$ denoting the intrinsic embedding function.

Finally, we define the neighborhood extended edge embedding as:

$$f_{ij}^0 = (h_i^0 + h_j^0) \cdot e_{ij}^0 \tag{5}$$

We conclude all the above processes as:

$$h_i^0, f_{ij}^0 = \text{Embedding Block}\left(z_i, z_j, e_{ij}\right), \quad j \in \mathcal{N}(i) \tag{6}$$

1.2 Output module

Following PaiNN, we update the scalar embedding and vector embedding of nodes with multiple gated equivariant blocks:

$$t_i^l = \mathrm{Dense}_{o_2}^l([\|W_{o_1}^l \vec{v}_i^l\|, h_i^l]) \tag{7}$$

$$h_i^{l+1} = W_{o_3}^l t_i^l \tag{8}$$

$$\vec{v}_i^{l+1} = W_{o_4}^l \vec{v}_i^l \odot W_{o_5}^l t_i^l \tag{9}$$

where $[\cdot,\cdot]$ is the tensor concatenation operation. The final scalar embedding $h_i^L \in \mathbb{R}^{N\times 1}$ and vector embedding $\vec{v}_i^L \in \mathbb{R}^{N\times 3\times 1}$ are used to predict various molecular properties.

On QM9, the molecular dipole is calculated as follows:

$$\mu = \left\|\sum_{i=1}^{N} \vec{v}_i^L + h_i^L(\vec{r}_i - \vec{r}_c)\right\| \tag{10}$$

where $\vec{r}_c$ denotes the center of mass. Similarly, for the prediction of electronic spatial extent $\langle R^2\rangle$, we use the following equation:

$$\langle R^2\rangle = \sum_{i=1}^{N} h_i^L \|\vec{r}_i - \vec{r}_c\|^2 \tag{11}$$

For the remaining 10 properties y, we simply aggregate the final scalar embedding of nodes as follows:

$$y = \sum_{i=1}^{N} h_i^L \tag{12}$$

For models trained on the molecular dynamics datasets including MD17, revised MD17, and MD22, the total potential energy is obtained as the sum of the final scalar embedding of the nodes. As an energy-conserving potential, the forces are then calculated using the negative gradients of the predicted total potential energy with respect to the atomic coordinates:

$$E = \sum_{i=1}^{N} h_i^L \tag{13}$$

$$\vec{F}_i = -\nabla_i E \tag{14}$$

1.3 ViSNet with Improper Angles

The ViS-MP with additional improper angles are shown as follows:

$$m_i^l = \sum_{j \in \mathcal{N}(i)} \phi_m^s \left(h_i^l, h_j^l, f_{ij}^l\right) \tag{15}$$

$$\vec{m}_i^l = \sum_{j \in \mathcal{N}(i)} \phi_m^v \left(m_{ij}^l, \vec{r}_{ij}, \vec{v}_j^l\right) \tag{16}$$

$$h_i^{l+1} = \phi_{un}^s \left(h_i^l, m_i^l, \langle \vec{v}_i^l, \vec{v}_i^l \rangle\right) \tag{17}$$

$$f_{ij}^{l+1} = \phi_{ue}^s \left(f_{ij}^l, \langle \mathrm{Rej}_{\vec{r}_{ij}}(\vec{v}_i^l), \mathrm{Rej}_{\vec{r}_{ji}}(\vec{v}_j^l)\rangle, \langle \mathrm{Rej}_{\vec{r}_{ij}}(\vec{v}_i^l), \mathrm{Rej}_{\vec{r}_{ji}}(\vec{v}_i^l)\rangle\right) \tag{18}$$

$$\vec{v}_i^{l+1} = \phi_{un}^v \left(\vec{v}_i^l, m_i^l, \vec{m}_i^l\right) \tag{19}$$

$\vec{f}_{ij}$ is updated by the inner product of the rejection of the vector embedding $\vec{v}_i$, $\vec{v}_j$ and $\vec{v}_i$ and $\vec{v}_i$ respectively through an update function ϕ_{ue}^s. Following the equation above, the residual edge embedding Δf_{ij}^{l+1} is computed by the Hadamard product of the runtime dihedral torsion and improper information with the transformed edge embedding:

$$\begin{aligned} \Delta f_{ij}^{l+1} = & \left\langle \mathrm{Rej}_{\vec{r}_{ij}}(W_{Rt1}^l \vec{v}_i^l), \mathrm{Rej}_{\vec{r}_{ji}}(W_{Rs1}^l \vec{v}_j^l)\right\rangle \odot \mathrm{Dense}_{\mathrm{Dihedral}}^l(f_{ij}^l) + \\ & \left\langle \mathrm{Rej}_{\vec{r}_{ij}}(W_{Rt2}^l \vec{v}_i^l), \mathrm{Rej}_{\vec{r}_{ji}}(W_{Rs2}^l \vec{v}_i^l)\right\rangle \odot \mathrm{Dense}_{\mathrm{Improper}}^l(f_{ij}^l) \end{aligned} \tag{20}$$

1.4 ViSNet with Transformer-M

Transformer-M is a powerful molecular model coping with 2D & 3D graphs. It is able to handle different molecular modalities sharing the same backbone architecture and alternatively activates their channels. By unified training across different views, Transformer-M preserves the essential knowledge and obtains molecular representations. Furthermore, it considers the 2D & 3D molecules to be fully connected and pads them with graph tokens. To preserve crucial graph features, it incorporates positional encoding including degree, shortest path, and distances in 3D conformers as learnable attention biases. With its powerful design and elaborate supervised signals, Transformer-M reveals its formidable capability for molecular representation learning across different data modalities. We combined the Transformer-M with the RGC module. With a more comprehensive and efficient geometric representation during training, it achieved better results than original Transformer-M and other methods, even without 3D information during the validation.

1.5 Variants of ViSNet

The refined ViS-MP mechanism of ViSNet is summarized as follows (decouple the message function and aggregation):

$$m_{ij}^l = \phi_{MS} \left(h_i^l, h_j^l, f_{ij}^l\right) \tag{21}$$

$$\vec{m}_{ij}^l = \phi_{MV} \left(m_{ij}^l, \vec{r}_{ij}, \vec{v}_j^l\right) \tag{22}$$

$$m_i^l = \sum_{j \in \mathcal{N}(i)} m_{ij}^l \tag{23}$$

$$\vec{m}_i^l = \sum_{j \in \mathcal{N}(i)} \vec{m}_{ij}^l \tag{24}$$

$$h_i^{l+1} = \phi_{US_N} \left(h_i^l, m_i^l, \langle \vec{v}_i^l, \vec{v}_i^l \rangle\right) \tag{25}$$

$$\vec{v}_i^{l+1} = \phi_{UV_N} \left(\vec{v}_i^l, m_i^l, \vec{m}_i^l\right) \tag{26}$$

$$f_{ij}^{l+1} = \phi_{US_E} \left(f_{ij}^l, \left\langle \mathrm{Rej}_{\vec{r}_{ij}}(\vec{v}_i^l), \mathrm{Rej}_{\vec{r}_{ji}}(\vec{v}_j^l)\right\rangle\right) \tag{27}$$

For *ViSNet-w/o A*, we exclude the runtime angle calculation and don't update the node embedding using inner product of vector embedding, i.e., the Eq. 25 is modified as:

$$h_i^{l+1} = \phi_{US_N} \left(h_i^l, m_i^l\right) \tag{28}$$

From the view of Vec2Scalar module, the residual node embedding Δh_i is computed as:

$$\Delta h_i^{l+1} = W_{\mathrm{Angle}}^l m_i^l + W_{\mathrm{res}}^l m_i^l \tag{29}$$

where m_i^l is directly projected through W_{Angle} and other modules remain the same.

For *ViSNet-w/o D*, we exclude the runtime dihedral calculation and don't update the node embedding using inner product of rejected vector embedding, i.e., the Eq. 27 is modified as:

$$f_{ij}^{l+1} = \phi_{US_E}\left(f_{ij}^l\right) \tag{30}$$

From the view of Vec2Scalar module, the residual node embedding Δf_{ij} is computed as:

$$\Delta f_{ij}^{l+1} = \text{Dense}_{\text{Dihe}}^l(f_{ij}^l) \tag{31}$$

where edge embedding f_{ij} is directly passed through a dense layer $\phi_{US_E}(\cdot)$ and other modules remain the same.

For *ViSNet-w/o A&D*, we exclude both angles and dihedrals information from ViSNet, and it becomes an invariant neural network which solely leverages scalar features, i.e., distance and atom types to predict the molecular properties.

For *ViSNet-N*, we exclude the edge embedding f_{ij} and directly aggregate the runtime dihedral into the intersection nodes instead of intersection edges. The paradigm for *ViSNet-N* can be modified by excluding the Eq. 27 and change Eq. 25 to:

$$h_i^{l+1} = \phi_{US_N}\left(h_i^l, m_i^l, \langle\vec{v}_i^l, \vec{v}_i^l\rangle, \left\langle \text{Rej}_{\vec{r}_{ij}}(\vec{v}_i^l), \text{Rej}_{\vec{r}_{ji}}(\vec{v}_j^l)\right\rangle\right) \tag{32}$$

From the view of Vec2Scalar module, the residual node embedding Δh_i is computed as:

$$\Delta h_i^{l+1} = \left\langle W_t^l\vec{v}_i^l, W_s^l\vec{v}_i^l\right\rangle \odot W_{\text{Angle}}^l m_i^l + \sum_{j\in\mathcal{N}(i)} \left\langle \text{Rej}_{\vec{r}_{ij}}(W_{Rt}^l\vec{v}_i^l), \text{Rej}_{\vec{r}_{ji}}(W_{Rs}^l\vec{v}_j^l)\right\rangle \odot W_{\text{Dihe}}^l m_i^l + W_{\text{res}}^l m_i^l \tag{33}$$

with runtime dihedral directly multiplying with scalar message m_i, and sum all three message features. Other modules remain the same.

For *ViSNet-T*, we design another form of dihedral calculation. In other words, we concentrate on the *vertex normal vectors*, intended as a replacement to the true geometric normal of the surface. Vertex normal vector, which is denoted as t in this paper, is computed as the normalized average of the surface normals of the faces that contain that vertex, given by:

$$\vec{t}_i = \sum_{j,k\in\mathcal{N}(i)} \text{NormalVec}(\text{Plane}_{jik}) \tag{34}$$

with j,k denoting the 1-hop neighbors of node i, $\sum(\cdot)$ denoting the reduction operation, NormalVec$(\cdot)$ standing for the calculation of the plane normal vector of the plane Plane_{jik}. It is noting that if all neighbors of node i participating in the vertex normal vector calculation, the rumtime complexity would increase to $O(|\mathcal{N}^2|)$. Therefore, to reduce the computational overhead, we only calculate the normal vector of the adjacent planes., i.e., only $\#\mathcal{N}(i)$ plane normal vectors are computed. # denotes the quantities. And the computational complexity can be reduced to $O(|\mathcal{N}|)$.

To be concrete, we first calculate the center of mass of the molecular graph denoting as $\vec{r}$.

$$\vec{r} = \frac{1}{N}\sum_{i=1}^{N} r_i \tag{35}$$

with N denoting the number of nodes in a molecular graph, r_i denoting the positions of node i.

Then vector $\vec{r}_i$ (pointing from center of mass to node i) is rotated to Z-axis as well as its 1-hop neighbors.

$$\mathcal{R}_x(\theta_x) = \begin{pmatrix} 1 & 0 & 0 \\ 0 & \cos\theta_x & -\sin\theta_x \\ 0 & \sin\theta_x & \cos\theta_x \end{pmatrix} \tag{36}$$

$$\mathcal{R}_y(\theta_y) = \begin{pmatrix} \cos\theta_y & 0 & \sin\theta_y \\ 0 & 1 & 0 \\ -\sin\theta_y & 0 & \cos\theta_y \end{pmatrix} \tag{37}$$

$$\vec{r}_j^{\,z} = \mathcal{R}_x(\theta_x)\cdot\mathcal{R}_y(\theta_y)\cdot\vec{r}_j \tag{38}$$

where θ_{xyz} represents the angles with XYZ-axis of node i, respectively and $\mathcal{R}$ denotes the rotation matrix. $j \in \mathcal{N}(i)$ is the 1-hop neighbors of node i.

We project the 1-hop neighbors to XOY plane and index them according to their angles with X-axis. The above method is able to ensure the rotational equivariance and permutational invariance when calculating the vertex normal vectors. After obtaining the sequential node index, we can compute the adjacent plane normal vectors, i.e., plane formed by 3 consecutive neighbors, and reduce them to node i. Similar to the angle calculation in our proposed RGC, the dihedral angles can be calculated through inner product:

$$\langle\vec{t}_i, \vec{t}_j\rangle = \sum_{m=1}^{N_i}\sum_{n=1}^{N_j} \cos\theta_{mijn} \tag{39}$$

with m, n denoting the neighbors of node i and node j.

It is noting that $\vec{t}$ is similar to $\vec{v}$ and be initialized to $\vec{0}$. We term $\vec{t}$ as normal vector embedding for brevity. The paradigm of *ViSNet-T* can be summarized as:

$$m_{ij}^l = \phi_{MS}\left(h_i^l, h_j^l, e_{ij}\right) \tag{40}$$

$$\vec{m}_{ij}^l = \phi_{MV}\left(m_{ij}^l, \vec{r}_{ij}, \vec{v}_j^l, \vec{t}_j^l\right) \tag{41}$$

$$m_i^l = \sum_{j \in \mathcal{N}(i)} m_{ij}^l \tag{42}$$

$$\vec{m}_i^l = \sum_{j \in \mathcal{N}(i)} \vec{m}_{ij}^l \tag{43}$$

$$h_i^{l+1} = \phi_{US_N}\left(h_i^l, m_i^l, \langle \vec{v}_i^l, \vec{v}_i^l \rangle, \langle \vec{t}_i^l, \vec{t}_i^l \rangle\right) \tag{44}$$

$$\vec{v}_i^{l+1}, \vec{t}_i^{l+1} = \phi_{UV_N}\left(\vec{v}_i^l, \vec{t}_i^l, m_i^l, \vec{m}_i^l\right) \tag{45}$$

$$\tag{46}$$

From the view of Scalar2Vec module, the vector messages $\vec{m}_{ij}^l$ is obtained by:

$$\vec{m}_{ij}^l = \left[\left(\text{Dense}_u^l(m_{ij}^l) \odot \vec{u}_{ij}\right) + \left(\text{Dense}_v^l(m_{ij}^l) \odot \vec{v}_{ij}^l\right), \left(\text{Dense}_t^l(m_{ij}^l) \odot \vec{t}_i^l\right)\right] \tag{47}$$

and the vector embedding $\Delta\vec{v}^l$ and normal vector embedding $\Delta\vec{t}^l$ are updated by:

$$\Delta\vec{v}_i^{l+1} = m_i^l + W_{\text{vm}}^l \vec{m}_i^l(v) \odot W_{\text{v}}^l \vec{v}_i^l \tag{48}$$

$$\Delta\vec{t}_i^{l+1} = m_i^l + W_{\text{tm}}^l \vec{m}_i^l(t) \odot W_t^l \vec{t}_i \tag{49}$$

with $\vec{m}_i^l(v)$ and $\vec{m}_i^l(t)$ denoting the first and second item in Eq. 47.

From the view of Vec2Scalar module, the node embedding h_i is updated by:

$$\Delta h_i^{l+1} = \left\langle W_t^l \vec{v}_i^l, W_s^l \vec{v}_i^l \right\rangle \odot W_{\text{Angle}}^l m_i^l + \left\langle W_t^l \vec{t}_i^l, W_s^l \vec{t}_i^l \right\rangle \odot +W_{\text{Dihe}}^l m_i^l W_{\text{res}}^l m_i^l \tag{50}$$

After all residual hidden representations are calculated, we add them with the original input of layer l and feed them to the next layer:

$$\begin{aligned} \vec{v}_i^{l+1} &= \vec{v}_i^l + \Delta\vec{v}_i^{l+1}, \\ \vec{t}_i^{l+1} &= \vec{t}_i^l + \Delta\vec{t}_i^{l+1}, \\ h_i^{l+1} &= h_i^l + \Delta h_i^{l+1} \end{aligned} \tag{51}$$

Compared with *ViSNet*, *ViSNet-T* is less elegant and neglects some dihedral information in order to reduce computational efficiency.

1.6 Further analysis

It is noting that even though *ViSNet-T* leverages the dihedral information through the inner product of $\vec{t}$, it still achieves worse performance than *ViSNet-w/o A*, *ViSNet-w/o D* and even *ViSNet-w/o A&D*. We deduce the reason why *ViSNet-T* is less effective is that vector embedding $\vec{v}$ and normal vector embedding $\vec{t}$ are represented in different embedding spaces. Simply adding two types of inner product embedding would confuse *ViSNet* to discriminate different structures in molecules.

1.7 Proofs of the equivariance of ViS-MP

Based on the evidence supporting the rotational invariance of runtime geometry calculation, we have additionally demonstrated that ViSNet exhibits rotational equivariance when producing vectors. Recall the Vector-Scalar interactive message passing (ViS-MP):

$$m_i^l = \sum_{j \in \mathcal{N}(i)} \phi_m^s \left(h_i^l, h_j^l, f_{ij}^l \right) \tag{52}$$

$$\vec{m}_i^l = \sum_{j \in \mathcal{N}(i)} \phi_m^v \left(m_{ij}^l, \vec{r}_{ij}, \vec{v}_j^l \right) \tag{53}$$

$$h_i^{l+1} = \phi_{un}^s \left(h_i^l, m_i^l, \langle \vec{v}_i^l, \vec{v}_i^l \rangle \right) \tag{54}$$

$$f_{ij}^{l+1} = \phi_{ue}^s \left(f_{ij}^l, \langle \mathrm{Rej}_{\vec{r}_{ij}}(\vec{v}_i^l), \mathrm{Rej}_{\vec{r}_{ji}}(\vec{v}_j^l) \rangle \right) \tag{55}$$

$$\vec{v}_i^{l+1} = \phi_{un}^v \left(\vec{v}_i^l, m_i^l, \vec{m}_i^l \right) \tag{56}$$

Equation 52 solely involves the scalar features invariant to SE(3) transformations. Equation 54 and 55 involve the scalar invariant features and RGC outputs, which have been proven to be rotational invariance. Therefore, our target is to prove the rotational equivariance of Equation 53 and 56.

Proofs. As shown in Methods Section, the implementation of Equation 53 is:

$$\vec{m}_{ij}^l = \left(\mathrm{Dense}_u^l(m_{ij}^l) \odot \vec{u}_{ij} \right) + \left(\mathrm{Dense}_v^l(m_{ij}^l) \odot \vec{v}_j^l \right) \tag{57}$$

Firstly, given the invariant scalar feature m_{ij}^l, $\mathrm{Dense}_u^l(m_{ij}^l)$ and $\mathrm{Dense}_v^l(m_{ij}^l)$ are also invariant after several dense layers. According to the PaiNN, the tensor product of scalar and vector features achieves rotational equivariance by scaling the vectors. Thus, given the arbitrary rotation matrix $R \in SO(3)$:

$$\left(\mathrm{Dense}_u^l(m_{ij}^l) \odot R\vec{u}_{ij} \right) + \left(\mathrm{Dense}_v^l(m_{ij}^l) \odot R\vec{v}_j^l \right) = R \left[\left(\mathrm{Dense}_u^l(m_{ij}^l) \odot \vec{u}_{ij} \right) + \left(\mathrm{Dense}_v^l(m_{ij}^l) \odot \vec{v}_j^l \right) \right] \tag{58}$$

$$= R\vec{m}_{ij}^l \tag{59}$$

We can subsequently obtain:

$$\sum_{j \in \mathcal{N}(i)} \phi_m^v \left(m_{ij}^l, R\vec{r}_{ij}, R\vec{v}_j^l \right) = R \sum_{j \in \mathcal{N}(i)} \phi_m^v \left(m_{ij}^l, \vec{r}_{ij}, \vec{v}_j^l \right) \tag{60}$$

$$= R\vec{m}_i^l \tag{61}$$

The implementation of Equation 56 is:

$$m_i^l = \sum_{j \in \mathcal{N}(i)} m_{ij}^l, \quad \vec{m}_i^l = \sum_{j \in \mathcal{N}(i)} \vec{m}_{ij}^l \tag{62}$$

$$\Delta \vec{v}_i^{l+1} = \vec{m}_i^l + W_{\mathrm{vm}}^l m_i^l \odot W_{\mathrm{v}}^l \vec{v}_i^l \tag{63}$$

The summation of $\vec{m}_{ij}^l$ preserves the rotational equivariance, i.e.,

$$\sum_{j \in \mathcal{N}(i)} R\vec{m}_{ij}^l = R\vec{m}_i^l \tag{64}$$

Next, W_{vm} and W_{v} are learnable matrix without bias. Thus the multiplication of W and $\vec{v}_i$ is the linear combination of equivariant vectors, which also ensures the rotational equivariance:

$$R\vec{m}_i^l + W_{\mathrm{vm}}^l m_i^l \odot W_{\mathrm{v}}^l (R\vec{v}_i^l) = R\vec{m}_i^l + W_{\mathrm{vm}}^l m_i^l \odot R(W_{\mathrm{v}}^l \vec{v}_i^l) \tag{65}$$

$$= R \left[\vec{m}_i^l + W_{\mathrm{vm}}^l m_i^l \odot W_{\mathrm{v}}^l \vec{v}_i^l \right] \tag{66}$$

$$= R \Delta \vec{v}_i^{l+1} \tag{67}$$

Similarly, we can prove the equivariance of Equation 56:

$$\phi_{un}^v \left(R\vec{v}_i^l, m_i^l, R\vec{m}_i^l \right) = R \left[\phi_{un}^v \left(\vec{v}_i^l, m_i^l, \vec{m}_i^l \right) \right] \tag{68}$$

$$= R\vec{v}_i^{l+1} \tag{69}$$

Once we have demonstrated the equivariance property of a single ViSNet block using layer l, we can infer inductively that this property is also preserved by the $1, 2, \ldots, L$ ViSNet blocks.

As part of our research efforts, we have focused on predicting atomic forces, which correspond to the gradient of conservative energy. In this regard, we have demonstrated the equivariance of the predicted forces from ViSNet. Specifically, we consider the ViSNet as a function $f(\cdot)$ that maps conformation coordinates $\mathcal{R}$ and atomic element types $\mathcal{Z}$ to the energy function. It is noteworthy that the interatomic distances $\mathbf{f}$ and corresponding angles θ, dihedral torsion φ, and improper potentials ψ are continuously differentiable with respect to the conformation coordinates. As previously observed in[?], the forces, i.e., gradients of conservative energy, can be backpropagated from these geometric features to coordinates through the following chain-rule procedure:

$$F_i = \frac{\partial \hat{E}}{\partial \mathcal{R}_i} = \frac{\partial f(\mathcal{Z}, \mathcal{R})}{\partial \mathcal{R}_i} \tag{70}$$

$$= \sum_{j \in N(i)} \frac{\partial f(\mathbf{f}, \theta, \varphi, \psi)}{\partial \left(f_{ij}, \langle \vec{v}_i, \vec{v}_i \rangle, \langle \text{Rej}_{\vec{r}_{ij}}(\vec{v}_i), \text{Rej}_{\vec{r}_{ji}}(\vec{v}_j) \rangle\right)} \cdot \frac{\partial \left(f_{ij}, \langle \vec{v}_i, \vec{v}_i \rangle, \langle \text{Rej}_{\vec{r}_{ij}}(\vec{v}_i), \text{Rej}_{\vec{r}_{ji}}(\vec{v}_j) \rangle\right)}{\partial \vec{r}_{ij}} \tag{71}$$

$$= \sum_{j \in N(i)} \mathbf{h} \cdot \frac{\partial \left(f_{ij}, \langle \vec{v}_i, \vec{v}_i \rangle, \langle \text{Rej}_{\vec{r}_{ij}}(\vec{v}_i), \text{Rej}_{\vec{r}_{ji}}(\vec{v}_j) \rangle\right)}{\partial \vec{r}_{ij}} \tag{72}$$

$$= \sum_{j \in N(i)} \mathbf{h} \cdot \frac{\partial R\vec{r}_{ij}}{\vec{r}_{ij}} \cdot \frac{\partial \left(f_{ij}, \langle R\vec{v}_i, R\vec{v}_i \rangle, \langle \text{Rej}_{R\vec{r}_{ij}}(R\vec{v}_i), \text{Rej}_{R\vec{r}_{ji}}(R\vec{v}_j) \rangle\right)}{\partial R\vec{r}_{ij}} \tag{73}$$

$$\tag{74}$$

where $\mathbf{h}$ is the scalar terms which is invariant to rotation and $\frac{\partial R\vec{r}_{ij}}{\vec{r}_{ij}}$ is the Jacobian matrix of $R\vec{r}_{ij}$ with respect to $\vec{r}_{ij}$, i.e., the transpose of rotation matrix R^T. It is worth noting that R is an orthogonal matrix, i.e., $R^{-1} = R^T$. Therefore, we can write the above equation as:

$$\frac{\partial \hat{E}}{\partial R \cdot \mathcal{R}_i} = \sum_{j \in N(i)} \mathbf{h} \cdot \frac{\partial \left(f_{ij}, \langle R\vec{v}_i, R\vec{v}_i \rangle, \langle \text{Rej}_{R\vec{r}_{ij}}(R\vec{v}_i), \text{Rej}_{R\vec{r}_{ji}}(R\vec{v}_j) \rangle\right)}{\partial R\vec{r}_{ij}} = R \cdot F_i \tag{75}$$

In a nutshell, the predicted atomic forces from ViSNet ensures rotational equivariance as well.

In the context of equivariance in high-order geometric tensors, it is worth noting that the rotational matrix R can be extended to the Wigner-D matrix D_l, with each block acting solely on a specific subspace.

1.8 Detailed settings for DFT and MM calculations

The replica exchange MD (REMD) simulations[1] utilized an initial structure sourced from the Protein Data Bank (PDB ID: 5AWL)[2]. Water molecules present in this crystal structure were excised. For the atomic interactions of Chignolin, we applied the FF19SB force field[3] within a generalized Born implicit solvent model. This model incorporated the second modification of the Bondi Van der Waals radii set[4]. To preserve chiral integrity at elevated temperatures during REMD simulations, we used the makeCHIR_RST tool in Amber 20[5] to generate a chiral restraint file.

The system underwent an energy minimization, consisting of 500 steepest descent cycles followed by 500 conjugate gradient cycles. This was succeeded by 200ps of equilibration runs across temperatures from 300K to 1000K, initialized with randomized velocities. The resultant structure from this equilibration was the base for REMD simulations at respective temperatures. In production, every replica ran for 2ps before exchanging with its adjacent temperature. This resulted in a total of 5,000 exchanges per production run, culminating in an 80 ns simulation over 8 replica temperatures. Sampling of the trajectory was done at 0.4ps intervals. The simulations were accomplished via Amber20 sander MPI version.

From the REMD trajectory, 10,000 uniformly distributed points were chosen to construct the input for Gaussian 16[6]. Each conformation's potential energy and atomic forces were determined using the M06-2X functional alongside the 6-31G* basis set, supported by a *superfine* precision integration grid. In summary, the Chignolin dataset incorporated 9,543 converged self-consistent field conformations, complete with total potential energy and atomic forces.

Calculations in molecular mechanics (MM) utilized the FF19SB force field[3]. We began by extracting target structures as PDB files. Using the tleap program, we generated the necessary topology and coordinate files. The sander program determined the energy of each structure and the force on individual atoms. Relative energies were derived by comparing each structure's energy to the one with the minimum energy.

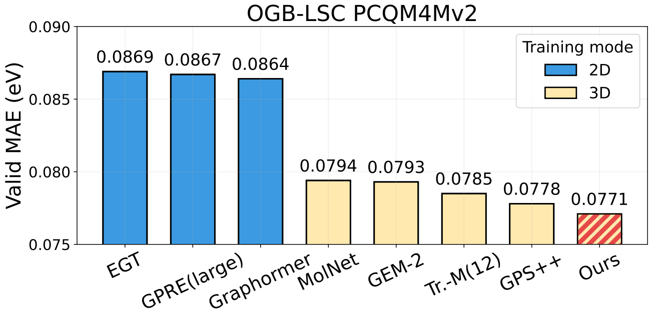

Figure S1. Mean absolute errors (MAE) of HOMO-LUMO gap (eV) on OGB-LSC PCQM4Mv2 valid set compared with state-of-the-art algorithms. The last bar with red shadow denotes the results of ViSNet. Source data are provided as a Source Data file.

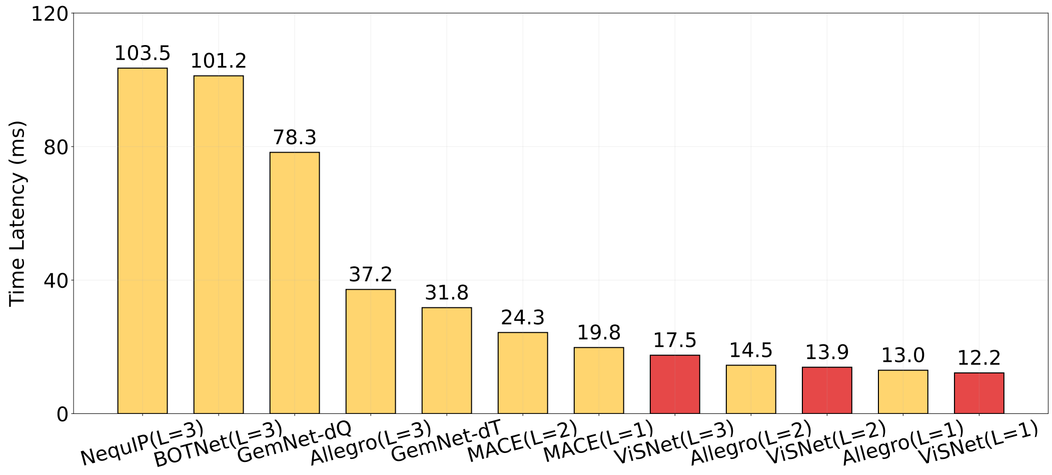

Figure S2. Comparison of time latency with current methods following MACE[7]. Time latency is defined as the time the model takes to compute forces on a structure. Experiments are conducted on a Nvidia A100 GPU. Source data are provided as a Source Data file.

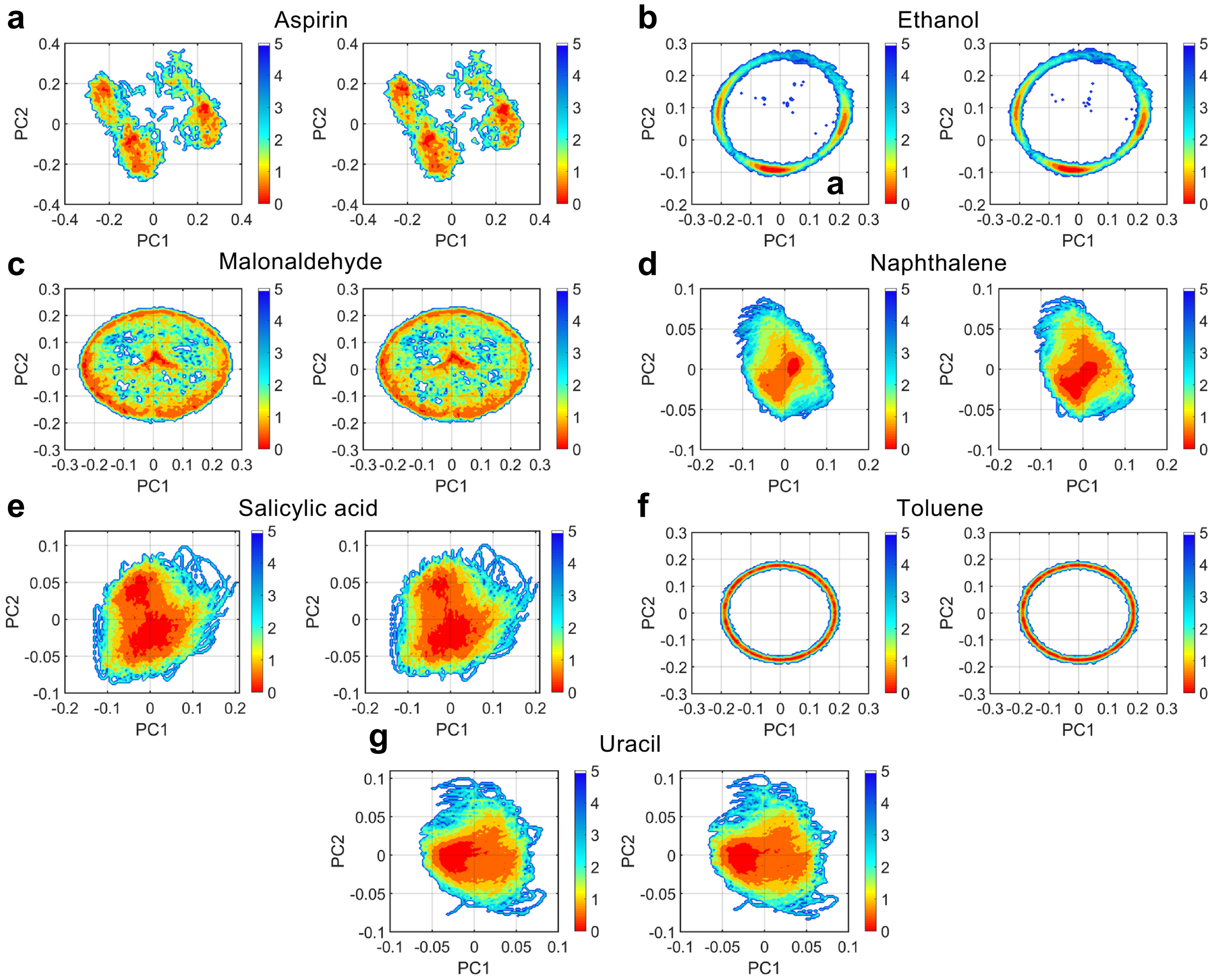

Figure S3. Potential energy surfaces (PES) explored by molecular dynamics simulation. Potential energy surfaces are plotted to inspect the conformational ensemble from simulations driven by DFT and ViSNet. The snapshots in each simulation trajectory are aligned to the initial structure of the corresponding molecule in MD17. Then, the principle component analysis (PCA) is applied on the coordinates for each conformations. PC1 and PC2 are set as two axes. The potential energy values were calculated as $\Delta G(x, y) = k_B T \ln g(x, y)$, where k_B is the Boltzmann constant, T is the temperature of systems and $g(x, y)$ represents the normalized joint probability distribution. The minimum energy value is set to zero. 100 bins are applied to generate the landscape in both x and y axis. **(a)** to **(g)** Potential energy surfaces (PES) for all seven organic molecules in MD17. In each panel, the left and right subfigures show the PES of the same molecule obtained by DFT and ViSNet, respectively. Source data are provided as a Source Data file.

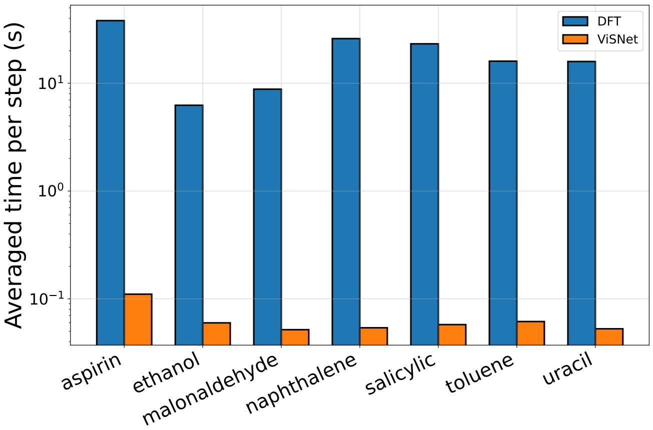

Figure S4. Time consumption of molecular dynamics simulations driven by ViSNet and DFT. Simulations are run with the ASE framework for the 7 molecules in MD17. The average time consumption for each simulation step is recorded in seconds. The y axis is a logarithmic axis with a base of 10. Source data are provided as a Source Data file.

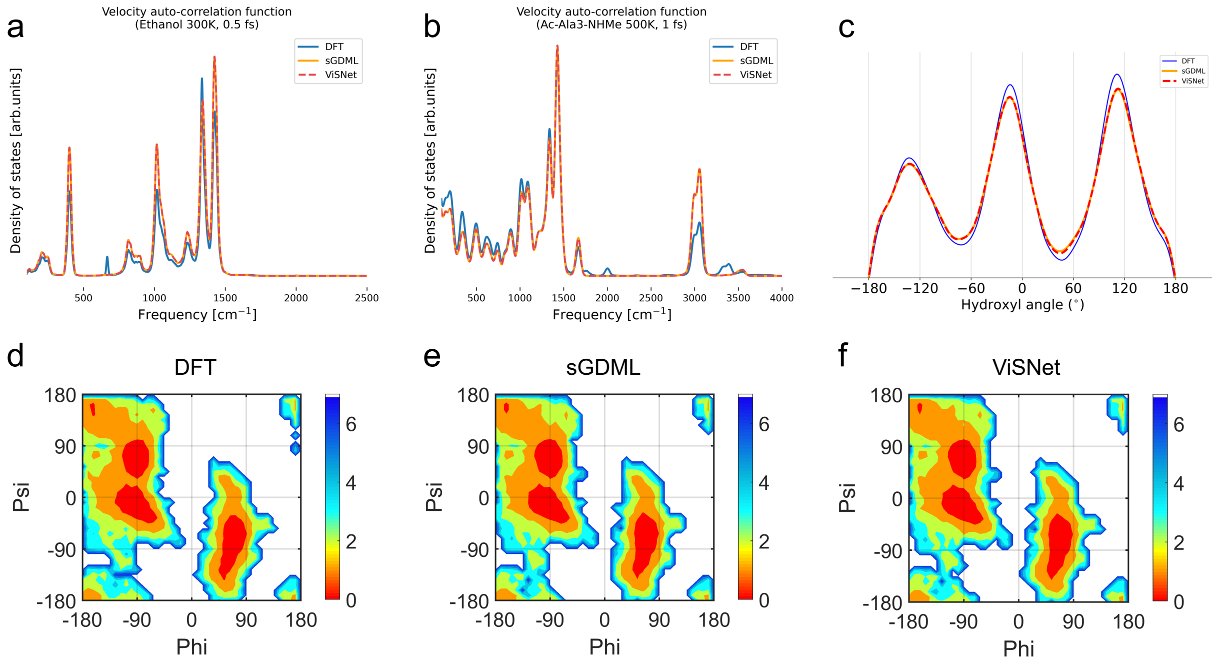

Figure S5. Analysis of molecular properties from MD simulations driven by DFT, sGDML and ViSNet. (a) Vibrational spectra analysis of Ethanol (MD17). **(b)** Vibrational spectra analysis of Ac-Ala3-NHMe (MD22). **(c)** Analysis of the probability distribution of hydroxyl angles in ethanol. **(d)** to **(f)** From left to right, the Ramachandran plots of Ac-Ala3-NHMe predicted or calculated by DFT, sGDML and ViSNet. The bar represents the values of free energy. Source data are provided as a Source Data file.

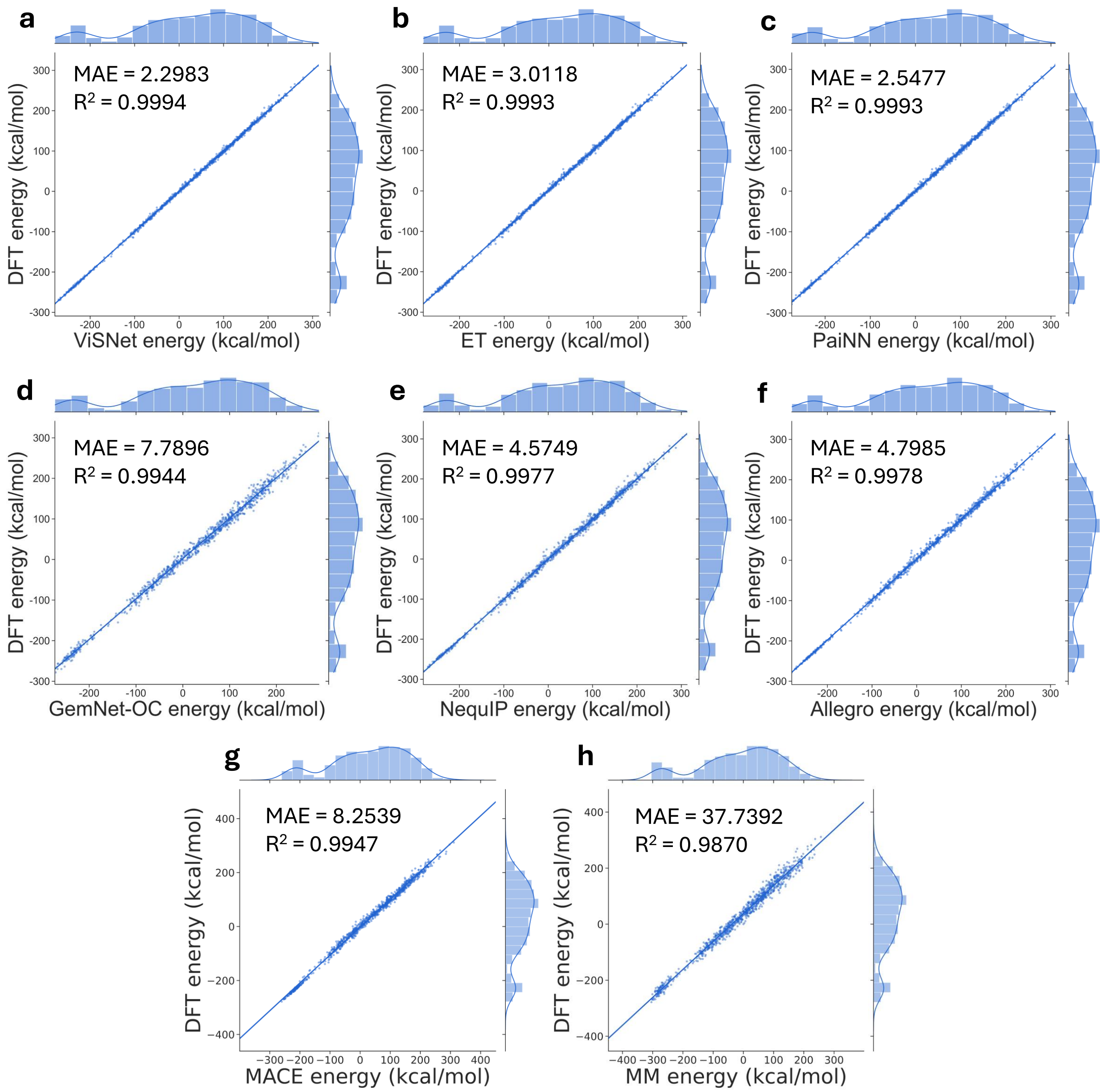

Figure S6. The energy correlations between the ground truth calculated by DFT and predictions or calculations by MLFFs and molecular mechanics (MM) respectively on the test dataset. (a) to **(h)** The results are predicted or calculated by ViSNet, ET, PaiNN, GemNet-OC, NequIP, Allegro, MACE and MM, respectively. The corresponding distributions of energy predictions or calculations as well as the ground truth are also shown in each panel. Source data are provided as a Source Data file.

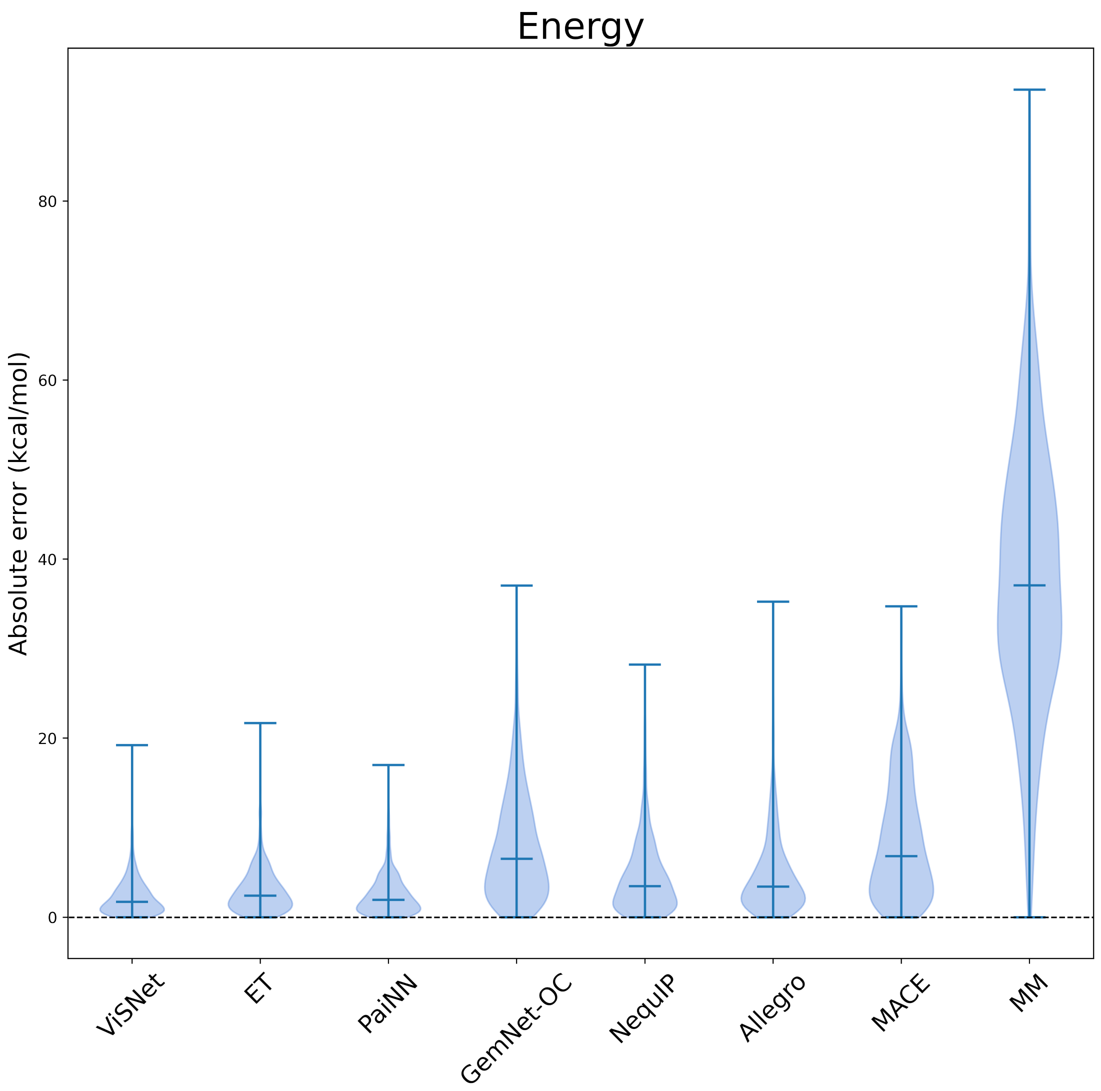

Figure S7. The violin plot of absolute errors between the ground truth calculated by DFT and predictions or calculations by MLFFs and molecular mechanics (MM) respectively on the test dataset. Source data are provided as a Source Data file.

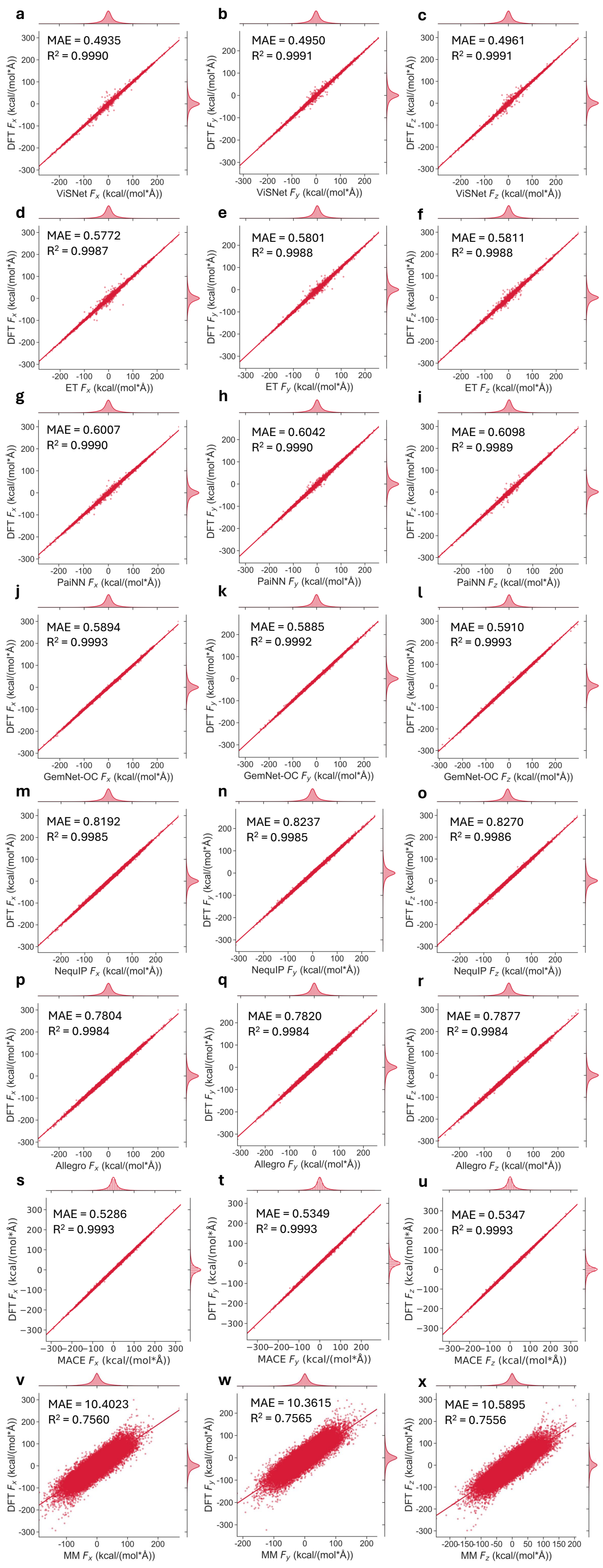

Figure S8. The forces correlations between the ground truth calculated by DFT and predictions or calculations by MLFFs and molecular mechanics (MM) respectively on the test dataset. (a) to **(x)** From top to bottom, the results are predicted or calculated by ViSNet, ET, PaiNN, GemNet-OC, NequIP, Allegro, MACE and MM, respectively. From left to right, the results are the component of forces in the three directions x, y, z, respectively. The corresponding distributions of forces predictions or calculations as well as the ground truth are also shown in each panel. Source data are provided as a Source Data file.

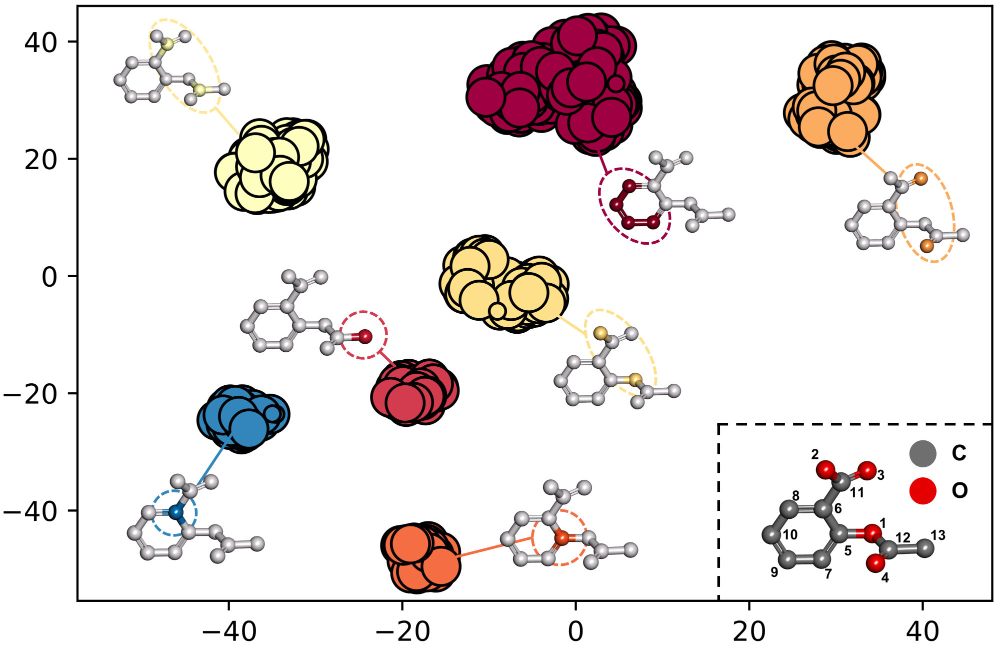

Figure S9. Visualization and model interpretability of ViSNet. Clusters of nodes' embeddings after the inner product of the direction units $\langle \vec{v}_i, \vec{v}_i \rangle$. The $\langle \vec{v}_i, \vec{v}_i \rangle$ represents angle representations with the intersecting node i as the vertex. The atoms in the chemical structure of aspirin corresponding to each cluster are colored with the same color of the cluster, while the remaining atoms are colored light gray. A chemical structure of Aspirin and the indices of atoms are illustrated in the bottom right region. Carbon and oxygen atoms are colored dark grey and red, respectively. The hydrogen atoms are omitted in both the clustering results and the chemical structure of aspirin for simplification.

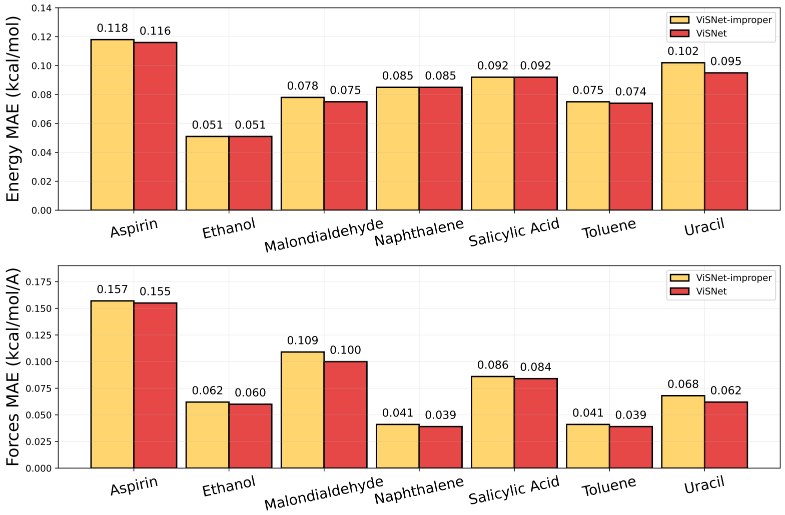

Figure S10. Comparison of results with improper angles on small organic molecules in MD17. ViSNet-improper achieves similar performance to ViSNet for small molecules, but exceeds that shown in Table 3 for large molecule in MD22. Source data are provided as a Source Data file.

Table S1. Glossary of notations

Notation	Description
$i; j; k; m; n$	Node (Atom) index in molecular graph
$\mathcal{N}(i)$	Neighborhood of node i
$l; L$	Index of layers; Number of layers
h_i	Node embedding of node i
$\phi(\cdot)$	Non-linear function or cosine cutoff
$\sum(\cdot)$	Reduce function
e_{ij}	Edge between node i and j
m_{ij}	Scalar message of node j and will be passed to node i
m_i	Scalar message of node i after aggregation
$\vec{m}_{ij}$	Vector message of node j and will be passed to node i
$\vec{m}_i$	Vector message of node i after aggregation
$\vec{r}_{ij}$	The relative position of node i,j
$\vec{u}_{ij}$	The unit direction from node i to node j
$\|\cdot\|$	Vector Norm
$\langle\cdot,\cdot\rangle$	Dot (Inner) product
$\text{Rej}_{\vec{u}_{ij}}$	Vecter rejection on vector $\vec{u}_{ij}$
z_i	Atom type of atom i
f_{ij}	Edge embedding
$\sigma(\cdot)$	Activation function
W	Learnable weight matrix
$\text{Dense}(\cdot)$	Dense layer
α_{ij}	Attention score in node i and node j
$\text{Attention}(\cdot)$	Attention mechanism
$\odot$	Hadamard product
Δ	Residual

Table S2. Mean and standard deviation of the time consumption of molecular dynamics simulations driven by ViSNet and DFT, respectively. The time consumption is averaged over ten parallel MD simulations.

Molecules	DFT speed	ViSNet speed	DFT speed std.	ViSNet speed std.
Aspirin	38.0977	0.1105	0.4434	0.0151
Ethanol	6.2474	0.0597	0.1639	0.0158
Malonaldehyde	8.8139	0.0517	0.2179	0.0027
Naphthalene	25.9500	0.0539	0.5022	0.0085
Salicylic Acid	23.2054	0.0577	0.2389	0.0131
Toluene	16.0254	0.0616	0.2277	0.0274
Uracil	15.9074	0.0527	0.3912	0.0035

Table S3. Ablation study of ViSNet on aspirin in MD17 dataset. The best results are shown in bold.

	energy	forces
ViSNet	**0.116**	**0.155**
ViSNet-improper	0.118	0.157
ViSNet-w/o A	0.121	0.174
ViSNet-w/o D	0.124	0.224
ViSNet-w/o A&D	0.289	0.654
ViSNet-N	0.123	0.224
ViSNet-T	0.136	0.281
ViSNet$_{l=1}$	0.119	0.193

Table S4. Hyperparameters for ViSNet trained on QM9, Molecule3D, OGB-LSC, MD17, rMD17, Chignolin and MD22

Hyperparameters	QM9	Molecule3D	OGB-LSC	MD17	rMD17	Chignolin	MD22
maximum epochs	1000	300	500	3000	3000	1000	3000
early stopping patience	150	30	20	600	600	100	600
init learning rate	1e-4	2e-4	2e-4	{2e-4, 3e-4, 4e-4}	2e-4	2e-4	{2e-4, 1e-4}
lr patience	15	5	-	30	30	10	30
lr decay factors	0.8	0.8	-	0.8	0.8	0.8	0.8
lr warmup steps	10000	5000	150000	1,000	1,000	3000	1000
batch size	32	512	256	4	4	4	{8, 4, 2}
no. layers L	9	9	12	9	9	6	9
hidden size F	512	256	768	256	256	128	256
cutoff	5.0	5.0	-	5.0	5.0	5.0	{4.0, 5.0}
forces / energy weight	-	-	-	0.95/0.05	0.95/0.05	0.95/0.05	0.95/0.05

Supplementary References

[1] Qi, R., Wei, G., Ma, B. & Nussinov, R. Replica exchange molecular dynamics: A practical application protocol with solutions to common problems and a peptide aggregation and self-assembly example. In *Peptide self-assembly*, 101–119 (Springer, 2018).

[2] Honda, S. *et al.* Crystal structure of a ten-amino acid protein. *Journal of the American Chemical Society* **130**, 15327–15331 (2008).

[3] Tian, C. *et al.* ff19sb: Amino-acid-specific protein backbone parameters trained against quantum mechanics energy surfaces in solution. *Journal of chemical theory and computation* **16**, 528–552 (2019).

[4] Onufriev, A., Bashford, D. & Case, D. A. Exploring protein native states and large-scale conformational changes with a modified generalized born model. *Proteins: Structure, Function, and Bioinformatics* **55**, 383–394 (2004).

[5] Case, D. A. *et al.* *Amber 2021* (University of California, San Francisco, 2021).

[6] Frisch, M. J. *et al.* Gaussian~16 Revision C.01 (2016). Gaussian Inc. Wallingford CT.

[7] Batatia, I., Kovács, D. P., Simm, G. N., Ortner, C. & Csányi, G. Mace: Higher order equivariant message passing neural networks for fast and accurate force fields. *arXiv preprint arXiv:2206.07697* (2022).
