# Supplementary material for: Enhancing geometric representations for molecules with equivariant vector-scalar interactive message passing
Source: Nat Commun. 2024 Jan 5;15:313. doi: 10.1038/s41467-023-43720-2 (PMC10770089; doi:10.1038/s41467-023-43720-2)
Supplement: Supplementary file 3 — Reporting Summary [file 41467_2023_43720_MOESM3_ESM.pdf]

nature portfolio

Corresponding author(s): Tong Wang, Bin Shao

Last updated by author(s): Oct 19, 2023

# Reporting Summary

Nature Portfolio wishes to improve the reproducibility of the work that we publish. This form provides structure for consistency and transparency in reporting. For further information on Nature Portfolio policies, see our Editorial Policies and the Editorial Policy Checklist.

## Statistics

For all statistical analyses, confirm that the following items are present in the figure legend, table legend, main text, or Methods section.

| n/a | Confirmed | |
|---|---|---|
| ☐ | ☒ | The exact sample size ($n$) for each experimental group/condition, given as a discrete number and unit of measurement |
| ☐ | ☒ | A statement on whether measurements were taken from distinct samples or whether the same sample was measured repeatedly |
| ☒ | ☐ | The statistical test(s) used AND whether they are one- or two-sided<br>*Only common tests should be described solely by name; describe more complex techniques in the Methods section.* |
| ☒ | ☐ | A description of all covariates tested |
| ☒ | ☐ | A description of any assumptions or corrections, such as tests of normality and adjustment for multiple comparisons |
| ☐ | ☒ | A full description of the statistical parameters including central tendency (e.g. means) or other basic estimates (e.g. regression coefficient) AND variation (e.g. standard deviation) or associated estimates of uncertainty (e.g. confidence intervals) |
| ☒ | ☐ | For null hypothesis testing, the test statistic (e.g. $F$, $t$, $r$) with confidence intervals, effect sizes, degrees of freedom and $P$ value noted<br>*Give P values as exact values whenever suitable.* |
| ☒ | ☐ | For Bayesian analysis, information on the choice of priors and Markov chain Monte Carlo settings |
| ☐ | ☒ | For hierarchical and complex designs, identification of the appropriate level for tests and full reporting of outcomes |
| ☒ | ☐ | Estimates of effect sizes (e.g. Cohen's $d$, Pearson's $r$), indicating how they were calculated |

*Our web collection on statistics for biologists contains articles on many of the points above.*

## Software and code

Policy information about availability of computer code

| | |
|---|---|
| Data collection | No software was used for data collection. |
| Data analysis | Experiments were done with Python with version 3.9.15, Pytorch with version 1.11.0, Pytorch Geometric with version 2.1.0 and Pytorch Lightning with version 1.8.0. The code used to reproduce our results is available at https://github.com/microsoft/ViSNet. Matplotlib and seaborn were used for plotting figures. |

For manuscripts utilizing custom algorithms or software that are central to the research but not yet described in published literature, software must be made available to editors and reviewers. We strongly encourage code deposition in a community repository (e.g. GitHub). See the Nature Portfolio guidelines for submitting code & software for further information.

## Data

Policy information about availability of data

All manuscripts must include a data availability statement. This statement should provide the following information, where applicable:

- Accession codes, unique identifiers, or web links for publicly available datasets
- A description of any restrictions on data availability
- For clinical datasets or third party data, please ensure that the statement adheres to our policy

The MD17, MD22 datasets are available at http://www.quantum-machine.org/gdml/data/npz.
The rMD17 dataset is available at https://archive.materialscloud.org/record/filefilename=rmd17.tar.bz2&record_id=466.

The QM9 dataset is available at https://deepchemdata.s3-us-west-1.amazonaws.com/datasets/molnet_publish/qm9.zip.
The Molecule3D dataset is available at https://github.com/divelab/MoleculeX/tree/molx/Molecule3D.
The OGB-LSC PCQM4Mv2 dataset is available at https://ogb.stanford.edu/docs/lsc/pcqm4mv2.
The Chignolin dataset used in this study is available at https://github.com/microsoft/ViSNet.

## Research involving human participants, their data, or biological material

Policy information about studies with human participants or human data. See also policy information about sex, gender (identity/presentation), and sexual orientation and race, ethnicity and racism.

| | |
|---|---|
| Reporting on sex and gender | N.A. |
| Reporting on race, ethnicity, or other socially relevant groupings | N.A. |
| Population characteristics | N.A. |
| Recruitment | N.A. |
| Ethics oversight | N.A. |

Note that full information on the approval of the study protocol must also be provided in the manuscript.

# Field-specific reporting

Please select the one below that is the best fit for your research. If you are not sure, read the appropriate sections before making your selection.

☒ Life sciences ☐ Behavioural & social sciences ☐ Ecological, evolutionary & environmental sciences

For a reference copy of the document with all sections, see nature.com/documents/nr-reporting-summary-flat.pdf

# Life sciences study design

All studies must disclose on these points even when the disclosure is negative.

Sample size

The MD17, rMD17, MD22, Molecule3D,OGB-LSC PCQM4Mv2 and QM9 datasets are widely used datasets in this field. We follow the same data split scheme in the previous studies [1-5]. The Chignolin dataset used in this study contains 9,543 samples sampled from Replica Exchange MD and calculated at DFT level which generally covers the whole potential energy space [6-7].


[1] Schütt, Kristof, Oliver Unke, and Michael Gastegger. "Equivariant message passing for the prediction of tensorial properties and molecular spectra." International Conference on Machine Learning. PMLR, 2021.
[2] Thölke, Philipp, and Gianni De Fabritiis. "Torchmd-net: equivariant transformers for neural network based molecular potentials." arXiv preprint arXiv:2202.02541 (2022).
[3] Xu, Zhao, et al. "Molecule3d: A benchmark for predicting 3d geometries from molecular graphs." arXiv preprint arXiv:2110.01717 (2021).
[4] Hu, Weihua, et al. "Ogb-lsc: A large-scale challenge for machine learning on graphs." arXiv preprint arXiv:2103.09430 (2021).
[5] Chmiela, Stefan, et al. "Accurate global machine learning force fields for molecules with hundreds of atoms." Science Advances 9.2 (2023): eadf0873.
[6] Wang, Tong, et al. "AIMD-Chig: Exploring the conformational space of a 166-atom protein Chignolin with ab initio molecular dynamics." Scientific Data , 2023, 10(1): 549.
[7] Wang, Zun, et al. "Improving machine learning force fields for molecular dynamics simulations with fine-grained force metrics." The Journal of Chemical Physics 159.3 (2023).


| | |
|---|---|
| Data exclusions | No data is excluded. |
| Replication | For datasets with relatively large amounts of data, we performed training only once and reported the results on the test/valid set. (A common practice in this field.) For smaller datasets, we initialized the model with 5 different random seeds and could get similar performance. For a pre-trained model, the exact same results could be obtained on the test set. |
| Randomization | The training, validation and test sets are in random and/or scaffold splits. |
| Blinding | During model training, the test set is blinded to models. |

# Reporting for specific materials, systems and methods

We require information from authors about some types of materials, experimental systems and methods used in many studies. Here, indicate whether each material, system or method listed is relevant to your study. If you are not sure if a list item applies to your research, read the appropriate section before selecting a response.

## Materials & experimental systems

| n/a | Involved in the study |
|---|---|
| ☒ | ☐ Antibodies |
| ☒ | ☐ Eukaryotic cell lines |
| ☒ | ☐ Palaeontology and archaeology |
| ☒ | ☐ Animals and other organisms |
| ☒ | ☐ Clinical data |
| ☒ | ☐ Dual use research of concern |
| ☒ | ☐ Plants |

## Methods

| n/a | Involved in the study |
|---|---|
| ☒ | ☐ ChIP-seq |
| ☒ | ☐ Flow cytometry |
| ☒ | ☐ MRI-based neuroimaging |
